# Lingonberry (*Vaccinium vitis-idaea* L.) Interact With *Lachnum pygmaeum* to Mitigate Drought and Promote Growth

**DOI:** 10.3389/fpls.2022.920338

**Published:** 2022-06-09

**Authors:** Hu Lou, Chao Guo, Baozhen Fan, Rao Fu, Heng Su, Jie Zhang, Long Sun

**Affiliations:** ^1^College of Life Science, Northeast Forestry University, Harbin, China; ^2^School of Forestry, Northeast Forestry University, Harbin, China; ^3^School of Resources and Environmental Science, Northeast Agricultural University, Harbin, China

**Keywords:** symbiosis, drought stress, photosynthetic pigment, Ericoid mycorrhiza, growth

## Abstract

The application of Ericoid mycorrhizal (ErM) fungi is considered to be an important strategy for increasing plant yield and drought resistance. In this study, we isolated and identified two ErM fungi that can promote the growth of lingonberry. We tried to understand the potential of these two ErM fungi to promote the growth of lingonberry and the strategies to help plants cope with water shortage. The use value of ErM fungi was evaluated by inoculating *Oidiodendron maius* FC (*Om*FC) or *Lachnum pygmaeum* ZL6 (*Lp*ZL6), well-watered (WW) and severe drought stress (SDS). The results showed that the mycelium of *Lp*ZL6 was denser than that of *Om*FC, and both ErM fungi significantly increased the biomass of lingonberry stems and roots. They also significantly increased the chlorophyll content by 65.6 and 97.8%, respectively. In addition, inoculation with *Lp*ZL6 fungi can improve drought resistance, promote root growth and increase root wet weight by 1157.6%. Drought reduced the chlorophyll content and soluble sugar content of lingonberry but increased significantly after inoculation with *Lp*ZL6. Inoculation with *Lp*ZL6 decreased lingonberry’s malondialdehyde (MDA) content but increased the superoxide dismutase (SOD) activity. Overall, these results indicated that the successful coexistence of ErM fungi and lingonberry alleviated the adverse effects of drought stress through higher secondary metabolites and photosynthetic pigment synthesis.

## Introduction

Lingonberry (*Vaccinium vitis-idaea* L.) is a low-shrub wild plant growing in the temperate and subtropical regions of the Northern Hemisphere. Its leaves and fruits have antibacterial, anticancer, and antioxidant effects (Lin et al., 2019). The berries are rich in vitamins (vitamins A, B, and C), calcium, magnesium, potassium, and phosphorus and have unique polyphenols and a large number of anthocyanins (Ek et al., 2006). Lingonberry juice not only has anti-inflammatory and anti-atherosclerotic properties (Kivimäki et al., 2012), but can also reduce blood sugar and lipids (Eid et al., 2014; Marungruang et al., 2020; Ryyti et al., 2020). Since *Vaccinium* plants usually lack root hairs, most plants are symbiotic with mycorrhizal fungi under natural conditions. The fungi associated with lingonberry are called Ericoid mycorrhizal (ErM) fungi (Lukesova et al., 2015). ErM fungi are beneficial microorganisms for the growth of lingonberry, which can promote the growth and nutrient absorption of blueberry and reduce the damage to plants under drought, heavy metals, and saline-alkali environments (Baba et al., 2021; Cai et al., 2021; Mu et al., 2021).

Ascomycota is the main fungus involved in the formation of ErM. *Oidiodendron maius* is a member of Leotiomycetes and has been isolated from *Rhododendron fortunei* (Wei et al., 2020). *Rhododendron fortunei* seedlings inoculated with the *Oidiodendron maius* strain produced abundant roots (Wei et al., 2016a), and significantly increased root and stem biomass (Wei et al., 2016b). *Lachnum pygmaeum* is a member of Pezizomycetes and has been isolated from *Vaccinium corymbosum* (Bizabani and Dames, 2015). Previous studies have shown that *Lachnum* sp. can form ErM fungi in plant roots, but it does not form a specific mycorrhizal structure. It is only characterized by mycelial infection in cortical cells, which cannot cause plant disease but can promote plant growth (Bizabani and Dames, 2015; Yang et al., 2018). However, another study showed that *Lachnum pygamaeum* and *Lachnum virgineum* formed a typical crimp ErM (Walker et al., 2011). This indicates that *Oidiodendron maius* and *Lachnum* spp. have the potential to form ErM structures, although their host adaptability and the environmental adaptability of plants have not been well proven.

Ericoid mycorrhizal plays an important role in promoting plant growth, which not only increases plant stem length and leaf area but also promotes plant root growth. The inoculation of ErM fungi can also alleviate the damage caused by environmental stress because it secretes secondary metabolites or produces specific symbiotic structures to help the host recruit beneficial bacteria and promote water absorption and plant growth and development (Cheng et al., 2021; El-Nashar et al., 2021; Sun et al., 2021). ErM plants have two reasons for their ability to effectively tolerate water deficit. First, fungal mycelia enter the plant root cells, and on the other hand, they have a wide area to absorb more water to plants (Smith et al., 2010; Doubková et al., 2013). Second, after the formation of ErM, fungi can promote plant physiological changes and limit the excessive production of reactive oxygen species (ROS) by signal transduction to enhance plant chlorophyll content, superoxide dismutase (SOD), catalase (CAT), peroxidase (POD), malondialdehyde (MDA) and antioxidant enzymes activity to reduce the damage of plant water deficit (Caravaca et al., 2005; Wu et al., 2014).

The economic benefit and ecological function bottleneck of lingonberry is due to the lack of ErM research (Daghino et al., 2016; Perotto et al., 2018; Vohník, 2020). In this paper, we reported the isolation and identification of mycorrhizal fungi from wild lingonberry in the Greater Khingan Range and analyzed the mycelium morphology and mycorrhiza structure after *in vitro* synthesis. In addition, we studied the effects of ErM inoculation on plant growth. Finally, we assessed the beneficial value of ErM fungi in helping lingonberry alleviate the water deficit.

## Materials and Methods

### Sample Collection, Isolation, and Pure Culture

**FIGURE 1:**
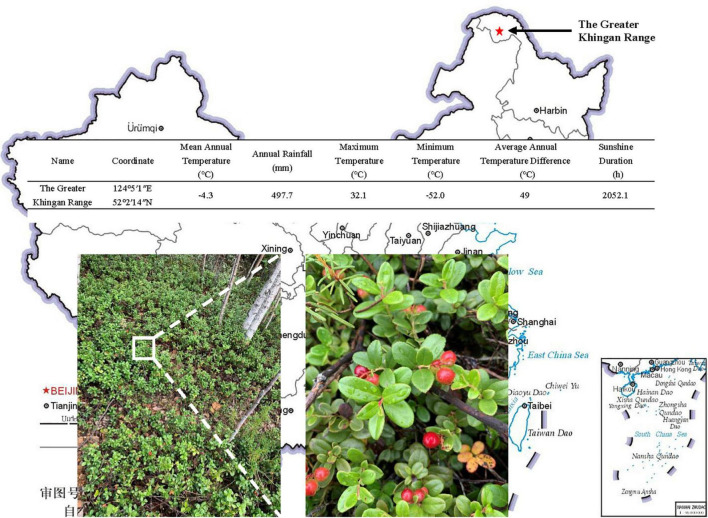
Location and climate information of The Greater Khingan Range.

The lingonberry was taken from The Greater Khingan Range, and the coordinates were 124°5′1″E, 52°2′14″N, Harbin, China (Figure 1). The samples were original undamaged woodlands, and the main tree species were *Larix gmelinii*, *Betula platyphylla*, *Pinus sylvestris var. mongolica*, *Populus davidiana*, etc. The main shrubs are *Rhododendron dauricum*, *Ledum palustre var. dilatatum*, *Vaccinium vitis-idaea*, etc. The main herbs are Cyperaceae, Compositae, Leguminosae, Rosaceae, etc., pH 4.85 ± 0.49. The soil layer has a very thick humus layer; the main litter pine needles and moss are widely distributed. The mean annual temperature is −4.3°C. The annual rainfall is 497.7 mm. Maximum temperature 32.1°C. Minimum temperature −52.0°C. The average annual temperature difference is 49°C. The sunshine duration is 2,052.1 h (Figure 1). Organic matter 105.96 ± 10.21 g kg^–1^, total phosphorus 1.09 ± 0.11 g kg^–1^, total nitrogen 11.30 ± 1.13 g kg^–1^, available phosphorus 73.64 ± 6.79 mg kg^–1^, available nitrogen 182.67 ± 15.68 mg kg^–1^ (Table 1).

**TABLE 1:** Soil chemical properties of lingonberry sampling site.

Name	pH	Organic matter (g kg^–1^)	Total phosphorus (g kg^–1^)	Total nitrogen (g kg^–1^)	Available phosphorus (mg kg^–1^)	Available nitrogen (mg kg^–1^)
The Greater Khingan Range	4.85 ± 0.49	105.96 ± 10.21	1.09 ± 0.11	11.30 ± 1.13	73.64 ± 6.79	182.67 ± 15.68

**FIGURE 2:**
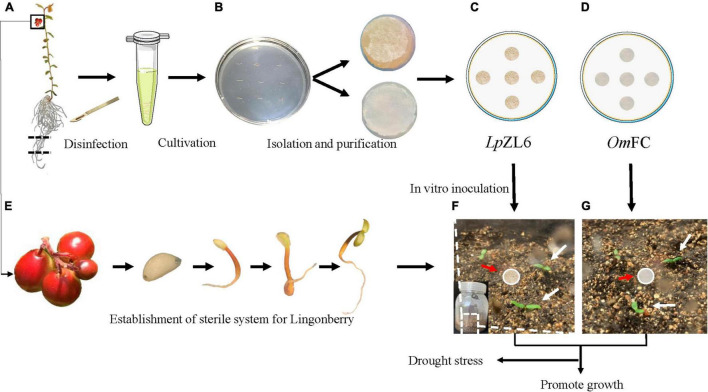
Obtaining aseptic seedlings of lingonberry and isolating ErM fungi. Disinfection of blueberry seedlings **(A)**, isolation of ErM fungi **(B)**, *Om*FC and *Lp*ZL6 fungi culture **(C,D)**, obtain aseptic seedlings of lingonberry **(E)**. Lingonberry inoculated with ErM fungi **(F,G)**.

The collected lingonberry root samples were stored at 4°C and sent back to the laboratory for mycorrhizal fungi isolation. The lingonberry roots were rinsed with flowing water for 5 min (Figure 2A) and then rinsed with sterilized water three times. Next, the washed roots were sterilized in 75% alcohol solution for 15 s and washed three times with sterilized water. Then, the roots were placed into 2% sodium hypochlorite disinfectant for 2 min and rinsed five times with sterilized water. The samples were dried on sterile filter paper in petri dishes for 10 min. The dried roots were cut into segments (approximately 5 mm long each) on PDA medium for 1 week at 25°C in darkness (Figure 2B). The fungus mycelium was separated and purified (Figures 2C,D). The structure of the mycelium was photographed by microscopy (OLYMPUS BX43) and scanning electron microscopy (HITACHI TM3030).

### DNA Extraction, Sequencing and Phylogenetic Analysis

Total DNA was extracted from fungi by the CTAB method (Murray and Thompson, 1980). PCR used 2 μL DNA, equivalent to 50 ng total DNA, with a final volume of 20 μL. The 20 μL reaction system contained 1 μL of template DNA, 4 pmol of each primer ITS1 and ITS4, 2 μL of dNTPs (2.5 mM dATP, dTTP, dCTP, and dGTP), 2 μL of 10× TransTaq HiFi Buffer II, 0.2 μL of TransTaq HiFi DNA Polymerase (www.transgen.com.cn, product code: AP131) and enough ddH_2_O to reach a total volume of 20 μL. Primers ITS1 and ITS4 are shown in Supplementary Table 1. ITS PCR products were sent to Bosch Biotechnology Co., Ltd. (Harbin, China) for DNA sequencing. ITS sequences were compared with those in the NCBI database by BLAST. The phylogenetic tree based on the ITS sequence was constructed by using MEGA 7.0 software and maximum likelihood method analysis (Saitou and Nei, 1987). Sequence data can be obtained from the NCBI database and submitted to GenBank with accession numbers ON032317.1 (*Om*FC) and ON007175.1 (*Lp*ZL6).

### Obtaining Aseptic Seedlings of Lingonberry and Synthesizing Mycorrhizae *in vitro*

Completely mature fruits were collected. The harvested lingonberry fruits were squeezed, and the mature seeds were stripped. The seeds were rinsed with clear water, dried, and placed in a 4°C refrigerator until use (Figure 2E). After a month, the seeds were sterilized in 75% alcohol solution for 30 seconds, washed three times with sterilized water, transferred to 2% sodium hypochlorite for 5 min, and washed five times with sterilized water. The samples were transferred to 1/2 MS medium supplemented with sucrose (30 g L^–1^) and agar (9 g L^–1^). The seedlings grew at low light intensity (30 μmol m^–2^s^–1^) in the culture room at 22 ± 2°C (Figure 2E). After 50 days, the lingonberry aseptic seedlings were planted in a sterile flask with soil: vermiculite = 2:1. The soil was put into the flask and cooled to room temperature after high-pressure sterilization. The seedlings were cultured at 22°C for two weeks under 16 h of light and 8 h of darkness for subsequent experiments (Figures 2F–J).

According to the method described by Wei et al. (2016a), *in vitro* mycorrhizal synthesis of *LpZL6* and *Om*FC fungi and lingonberry was carried out and slightly modified. We used the staining method developed by Upson et al. to further investigate the morphology of hyphae curl and the identification of hyphae colonization in root cells (Upson et al., 2007). The simple step is to first clean the roots in FAA for 24 h. Then, the samples were rinsed with sterile water, soaked in 10% KOH solution for 60 min and kept in 90°C water. After removal, it was washed with distilled water until colorless, placed in 5% lactic acid solution for 4 min, and placed in trypan blue dye solution at room temperature overnight. Finally, the root segments were removed, soaked in glycerol lactate and observed under a microscope.

### Study on the Growth-Promoting Activities of OmFC and LpZL6

The phosphorus utilization of fungi was measured by the P-V-Mo yellow colorimetric method. Fungi were cultured in an inorganic phosphorus liquid medium at 28°C for 5 days at 150 rpm. The supernatant fermentation broth (0.4 ml) was added to 2% citric acid solution (0.4 ml) and vanadium ammonium molybdate solution (4 ml), with deionized water volume to 10 ml after mixing. After standing at room temperature for 20 min, the absorbance of the sample at 456 nm wavelength was measured by UV spectrophotometer, and the content of available phosphorus in the fermentation broth was calculated according to the phosphorus standard curve. Fermentation broth samples were taken every 48 h, with three replicates per group.

The fungi were inoculated into liquid medium containing tryptophan and cultured at 28°C for 5 days at 150 rpm min^–1^. The indole-3-acetic acid (IAA) content in the fermentation broth was determined by Salkowski reagent. The operation was as follows: The supernatant was mixed with Salkowski reagent at a ratio of 1:2, and the sample was placed in the dark for 30 minutes. The absorbance of the sample at 530 nm was measured by a UV spectrophotometer, and the IAA content was calculated by an IAA standard curve. Fermentation broth samples were taken every 48 hours with 3 replicates per group.

### Determination of Growth Parameters and Related Physiological Indexes of Lingonberry

Germ-free plants were grown in the culture room of Northeast Forestry University (45°43′45.71″ northern latitude, 126°38′11.04″ east longitude, Heilongjiang Province, China). The lingonberry was inoculated with *Om*FC, *Lp*ZL6, and no fungi in a germ-free tissue culture bottle. The inoculation process was to bury the activated fungus piece at 0.5 cm deep in the middle of the aseptic seedlings of lingonberry (Figures 2F,G). Drought stress experiments included well-watered (WW) 100% field capacity (FC), severe drought stress (SDS) 50% FC and inoculation of ErM fungi. The design included the following treatments: WW and plants without fungal inoculation, SDS and plants without fungal inoculation, SDS and inoculated ErM fungal plants and WW inoculated ErM fungal plants. The soil water content was examined every 2 days through soil quality change for 60 days. The seedlings grew in the culture room at 22 ± 2°C. The experiment was carried out in a 16/8-h light/dark mode under greenhouse conditions with a light intensity of 30 μmol m^–2^ s^–1^. Statistical analysis and physiological indexes were measured after 60 days.

Measurements of chlorophyll content were performed according to the method described by Lichtenthaler (1987), and photosynthetic pigments (carotenoids and chlorophyll) were extracted from fresh leaves with 80% acetone. After centrifugation at 6,000 rpm for 15 min, the absorbance of the supernatant was recorded by a spectrophotometer at 470,646 and 661 nm. Total soluble sugar (TSS) measurements were performed according to the method of Masuko et al. (2005). Dry leaves (0.1 g) were ground with 12.5 ml ethanol and centrifuged at 8000 rpm for 10 min. The TSS was estimated at 490 nm by the phenol-sulfate acid method using enzyme calibration (BioTek ELx808, United States). The TSS content was calculated by a standard curve. The proline content was measured by the ninhydrin colorimetric method described by Bates et al. (1973), and the absorbance at 520 nm was measured. The proline concentration was calculated using a standard curve.

Measurement of MDA content in leaves Referring to Velikova et al. (2000), 0.5 g leaves were ground into homogenate in 5 ml 0.1% (w v^–1^) trichloroacetic acid (TCA), centrifuged at 11,500 × *g* for 10 min. One milliliter supernatant was mixed with 4 ml thiobarbituric acid (TBA) reagent (0.5% TBA in 20% TCA). The samples were heated at 95°C for 30 min, cooled rapidly in an ice bath and centrifuged at 11,500 × *g* for 15 min. The amount of MDA-TBA complex was measured by spectrophotometry at 532 and 600 nm, and the MDA content was calculated. SOD activity was measured by the method of Monazzah et al. (2018) and Sairam et al. (2002). The reaction solution was composed of 40 mmol L^–1^ phosphate buffer (pH 7.8), 0.1 mmol L^–1^ EDTA, 75 lmol L^–1^ NBT, 2 lmol L^–1^ riboflavin, 13 mmol L^–1^ methionine, and tissue extract. The absorbance of the mixture was measured by a spectrophotometer at 560 nm. One unit of SOD activity was defined as the amount of enzyme required for 50% nitroblue tetrazole (NBT) photoreduction inhibition. SOD activity is expressed as the enzyme activity of fresh weight per gram leaf.

### Statistical Analysis

All experiments were completely randomized and repeated four times. In the weight measurement, the weight of 10 plants was counted randomly. The root and bud were separated, and the bud fresh weight (FW) was immediately reported. Then, the dry weight (DW) of branches was measured after drying in an oven (70°C, 48 h). We conducted a *t-test* to determine the significant difference (*p* < 0.05 or *p* < 0.01, depending on the experiment). In root length measurements, we used six plants for counting. SPSS software v 19.0 was used to analyze the data.

## Results

### Colony Morphology, Systematic Development and Mycorrhizal Formation

**FIGURE 3:**
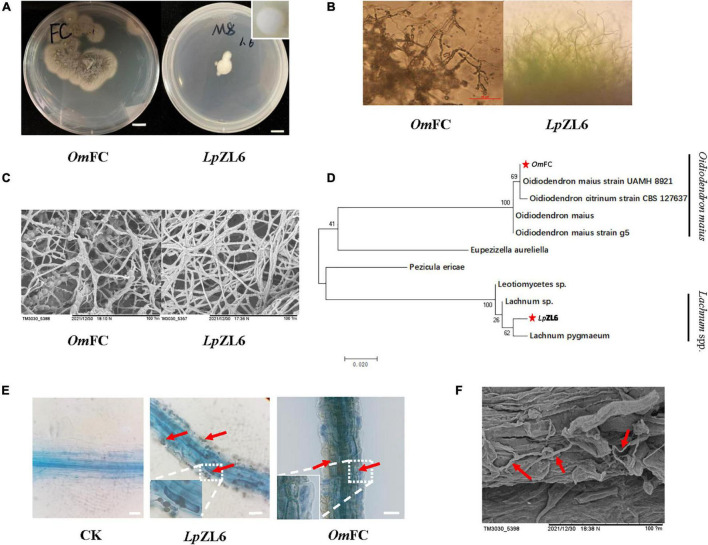
Fungi morphology and mycorrhizal synthesis. Identification of ErM fungi **(A–C)**, phylogenetic analysis of ErM fungi **(D)**, and ErM fungi inoculated plant root **(E,F)**.

In this study, two mycorrhizal fungi were isolated and purified from the roots of lingonberry in The Greater Khingan Range, and the colony morphology after isolation and purification was consistent with the identification results. The *Om*FC was irregular in shape and gradually changed from white to gray-brown dull, and intact in the early stage. No aerial mycelium was produced within two weeks. *Lp*ZL6 showed regular white mycelium, no luster, transparent, and dry texture, and the edge of the mycelium was grainy (Figures 3A,B). Scanning electron microscopy showed that *Lp*ZL6 mycelium was denser than *Om*FC (Figure 3C). The phylogenetic tree showed that *Om*FC belonged to the *Oidiodendron maius* evolutionary branch (NCBI accession number: ON032317.1), while *Lp*ZL6 belonged to *Lachnum* spp. evolutionary branch (NCBI accession number: ON007175.1). However, several characteristics between the two branches were different (Figure 3D). *Lp*ZL6 grew slowly and formed regular colonies and was 10.43 ± 0.17 mm 7 day^–1^ on PDA medium. In contrast, *Om*FC grew faster and formed irregular colonies and was 19.76 ± 2.81 mm 7 day^–1^ on PDA medium.

*Om*FC and *Lp*ZL6 were inoculated with lingonberry and were healthy before plant measurement. Lingonberry without fungal inoculation showed no fungal structure. All fungi colonized the fine roots of lingonberry seedlings and formed hyphae of typical ErM mycorrhiza fungi in important root cells (Figure 3E). The results showed that *Om*FC and *Lp*ZL6 successfully colonized the roots of lingonberry. Scanning electron microscopy showed hyphal colonization in the roots of lingonberry (Figure 3F).

### OmFC and LpZL6 Promote Plant Growth

**FIGURE 4:**
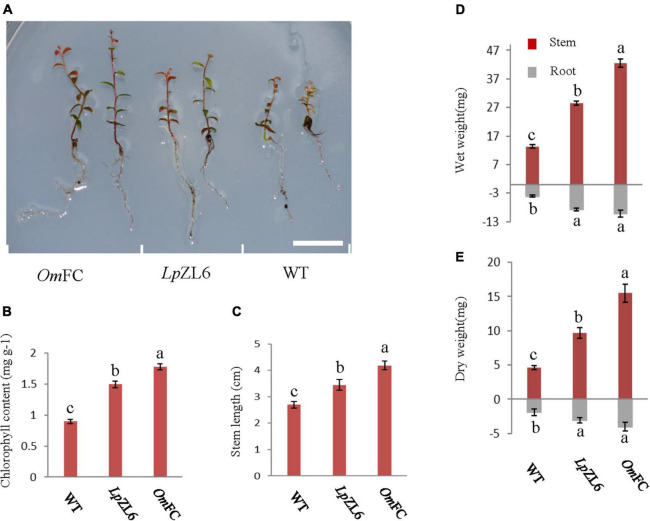
*Om*FC and *Lp*ZL6 promote plant growth. Inoculated *Om*FC and *Lp*ZL6 to lingonberry seedlings **(A)**. Inoculated *Om*FC and *Lp*ZL6 fungal chlorophyll content changes **(B)**. Changes in stem length of inoculated *Om*FC and *Lp*ZL6 fungi **(C)**. Inoculation with *Om*FC and *Lp*ZL6 increased the wet and dry weight of lingonberry stems and roots **(D,E)**. (Scale bar in a = 1 cm).

The growth test of lingonberry that evaluated the benefits of ErM fungal inoculation revealed significant differences between individual groups. Compared with no fungi inoculation, it has higher stemmed, more lush leaves and greener colors, *Om*FC and *Lp*ZL6 significantly increased the biomass of lingonberry (Figure 4A). It has higher stemmed, more lush leaves and greener colors. However, plants inoculated with *Om*FC (4.18 ± 1.39 cm) performed better than those inoculated with *Lp*ZL6 (3.45 ± 0.2 cm), and the stem length increased by 54.8 and 27.8% compared with WT, respectively (Figure 4C). The chlorophyll content of lingonberry inoculated with *Om*FC (1.78 ± 0.05 mg g^–1^) and *Lp*ZL6 (1.49 ± 0.06 mg g^–1^) increased by 97.8 and 65.6% compared with WT, respectively (Figure 4B). This confirms that the fungi *Om*FC and *Lp*ZL6 make lingonberry beneficial fungi and promote growth and chlorophyll accumulation.

ErM fungi were inoculated into the roots of lingonberry, and the results showed that *Om*FC and *LpZL6* could significantly promote the biomass of lingonberry plants. The stem wet weight of lingonberry inoculated with *Om*FC (42.53 ± 0.17 mg) and *Lp*ZL6 (28.56 ± 0.76 mg) increased by 217.8 and 113.5%, respectively, compared with that of lingonberry without inoculation. The stem dry weight of lingonberry inoculated with *Om*FC (15.5 ± 1.29 mg) and *Lp*ZL6 (9.71 ± 0.78 mg) increased by 236.2 and 110.6%, respectively, compared with that of lingonberry without inoculation (Figure 4D). Inoculation with ErM fungi not only increased the biomass of aboveground stems but also increased the biomass of roots. lingonberry had more and longer roots. The root wet weight of lingonberry inoculated with *Om*FC (10.26 ± 1.18 mg) and *Lp*ZL6 (8.75 ± 0.49 mg) increased by 154.6 and 117.1%, respectively, compared with that of non-inoculated fungi. The root dry weight of lingonberry inoculated with *Om*FC and *Lp*ZL6 was 113.2 and 61.9% higher than that of non-inoculated fungi, respectively (Figure 4E). These results indicated that inoculation with *Om*FC and *Lp*ZL6 promoted the accumulation of wet and dry weight of lingonberry.

### OmFC and LpZL6 Have the Characteristics of Producing IAA and Phosphate Solubilization

**FIGURE 5:**
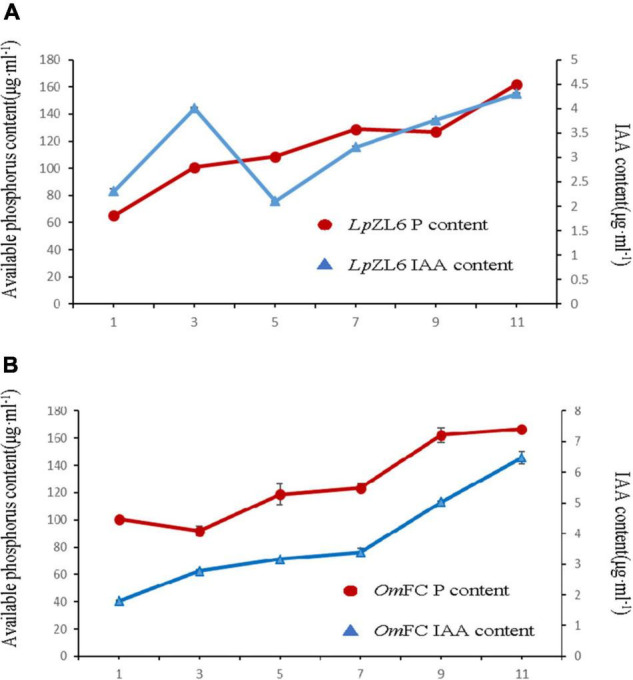
*Om*FC and *Lp*ZL6 have the characteristics of producing IAA and phosphate solubilization. 0–11 days *Lp*ZL6 to producing IAA and phosphate solubilization **(A)**. 0–11 days *Om*FC to producing IAA and phosphate solubilization **(B)**.

Studies suggest that the ability of fungi to produce IAA and phosphate solubilization is positively correlated with plant growth. To explore the growth-promoting ability of fungi, we analyzed IAA content and phosphate solubilization in mycelia of *Om*FC and *Lp*ZL6. The results showed that phosphate solubilization increased gradually from the first day to the eleventh day after inoculation with *Lp*ZL6, while phosphate solubilization first decreased then increased after inoculation with *Om*FC (Figure 5A). The IAA content produced by *Lp*ZL6 inoculation reached a high peak on the third day but began to decline on the fifth day and then gradually increased. IAA increased gradually from the first day to the eleventh day after inoculation with *Om*FC fungi (Figure 5B). *Om*FC and *Lp*ZL6 no significant difference in phosphate solubility.

### Inoculation With LpZL6 Promoted the Growth of Lingonberry and Alleviated Drought Stress Damage

**FIGURE 6:**
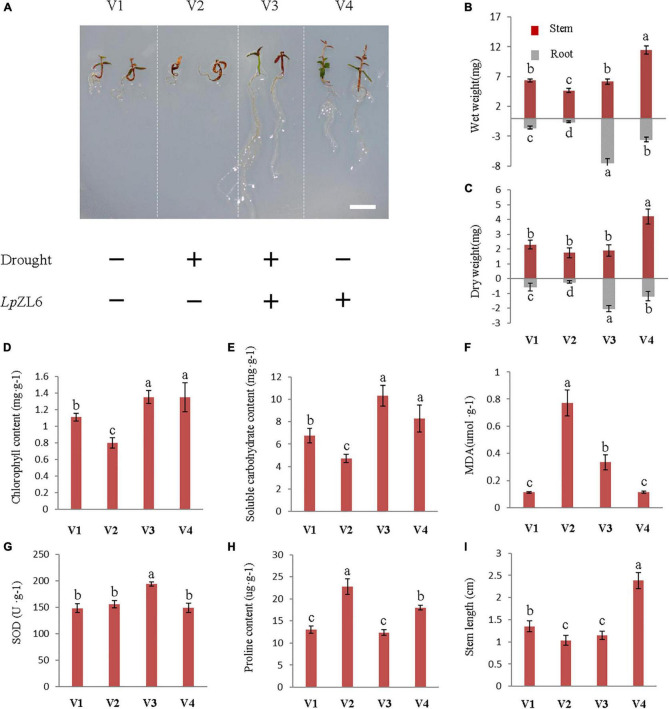
Inoculation with *Lp*ZL6 promoted the growth of lingonberry and alleviated drought stress damage. V1: Wild type; V2: + Drought; V3: + Drought + *Lp*ZL6; V4: + *Lp*ZL6. Drought stress inoculated with *Lp*ZL6 lingonberry seedlings **(A)**. Drought stress inoculated with *Lp*ZL6 fungi increased the wet weight and dry weight of lingonberry stems and roots **(B,C)**. Changes in chlorophyll content of *Lp*ZL6 inoculated fungi **(D)**. Changes in TSS content of *Lp*ZL6 inoculated fungi **(E)**. Changes in MDA content of *Lp*ZL6 inoculated fungi **(F)**. Changes in SOD activity of *Lp*ZL6 inoculated fungi **(G)**. Changes of proline content in *Lp*ZL6 fungi **(H)**. Changes of stem length of *Lp*ZL6 inoculated fungi **(I)**. [Scale bar in panel **(A)** = 0.5 cm].

Under drought stress, the biomass of roots and stems of lingonberry plants inoculated with ErM fungi increased compared with those of non-inoculated plants (Figure 6A). The stem wet weight (6.17 ± 0.45 mg) and root wet weight (7.42 ± 0.63 mg) of lingonberry inoculated with *Lp*ZL6 were increased by 32.1% and 1157.6%, respectively (Figure 6B). Interestingly, the development of inoculated *Lp*ZL6 fungal roots is exciting. Water deficit stress not only significantly reduced the fresh weight of stems and roots but also reduced the dry weight of stems and roots (*p* < 0.001). SDS reduced stem dry weight and root dry weight by 8.6 and 741.7%, respectively, compared with WW (Figure 6C), and non-inoculated ErM fungi lingonberry seedlings even died directly. These results showed that under the condition of water deficit, inoculation with *Lp*ZL6 fungi could not only alleviate the damage caused by drought stress on lingonberry seedlings but also significantly promote the growth and development of roots. The above results indicated that inoculation with *Lp*ZL6 fungi could promote root development and alleviate the damage caused by the water deficit in lingonberry. However, *Om*FC did not alleviate the water deficit.

Statistical analysis showed that drought stress significantly affected the chlorophyll content (*p* < 0.001). Compared with WW, the total chlorophyll concentration of non-inoculated plants (0.8 ± 0.06 mg g^–1^ FW) grown under SDS decreased by 38.8%, but inoculation with *Lp*ZL6 fungi (1.35 ± 0.08 mg g^–1^ FW) increased by 21.6% (Figure 6D). This indicated that the symbiosis between lingonberry and *Lp*ZL6 fungi alleviated the negative impact of drought stress. Variance analysis showed that under drought stress, *Lp*ZL6 inoculation and non-inoculation had significant effects on proline and TSS content (Figures 6E,H). The proline content of *Lp*ZL6-inoculated plants was significantly lower than that of non-inoculated plants. Under drought stress, the proline content of lingonberry inoculated with *LpZL6* fungi decreased by 84.1% compared with the control. In contrast, the TSS content was significantly increased by 119.5% compared with non-inoculated *Lp*ZL6 fungi (Figure 6H).

In this study, *Lp*ZL6 fungal inoculation significantly increased TSS in lingonberry leaves (Figure 6E) by 119.5% under drought stress. Therefore, water deficiency can lead to the accumulation of soluble carbohydrates in plants. Under drought stress, compared with non-inoculated fungi, the MDA content of *Lp*ZL6 fungi decreased by 57.1% (Figure 6F). Mycorrhizal inoculation led to an increase in SOD activity in plant tissues, which was 19.8% higher than that without *Lp*ZL6 inoculation (Figure 6G).

## Discussion

*Vaccinium* berries are widely praised worldwide for their medical value. Different countries have conducted systematic investigations on lingonberry berries, such as the United States (Grace et al., 2014), Canada (Kalt et al., 2008), Italy (Prencipe et al., 2014), and Finland (Latti et al., 2011). The results showed that wild lingonberry berries obviously had higher anthocyanin content and antioxidant activity than their varieties (Feng et al., 2016). Our experimental material lingonberry (Figure 1) is a red berry, also known as mountain cranberry, cowberry. Sweet and sour taste, leaves and fruits can treat urethritis, cystitis, enteritis, dysentery and other diseases (Kowalska, 2021; Parnanen et al., 2021; Rohrl et al., 2021). These berries are mainly developed and utilized in the United States and European countries. In contrast, there are many kinds of wild berry resources in China, and there are few reports. The natural distribution area of wild berries in Northeast China is the largest, approximately 15 million hectares, with the largest number of berries, especially Rhododendron. In addition, most of these berries are medicinal species (Feng et al., 2016). Therefore, research on ErM fungi and lingonberry roots can develop the cultivation ability of wild lingonberry in Northeast China on a large scale and improve the economic benefits of lingonberry.

Lingonberry mycorrhizal fungi are usually diverse. Studies have shown that *Lachnum* spp. is the dominant species in specific habitats, and specific colonization occurs on a certain type of plant (Korkama-Rajala et al., 2008). By estimating the diversity of mycorrhizal fungi in two herbaceous plants, two ascomycetes, *Hymenoscyphus* sp. and *Lachnum* sp. without mycorrhizal structure were found in mycorrhizal fungi, indicating that *Lachnum* spp. may also coexist with mycorrhizal fungi (Gao and Yang, 2010). Other studies have found that *Lachnum* sp. can colonize the root but does not form any specific mycorrhizal structure. Only mycelial infection in cortical cells is characterized by intracellular and intercellular colonization, which has no pathogenic effect on plants and can promote the growth of plant branches (Bizabani and Dames, 2015). However, another study found that *Lachnum* sp. was symbiotic with other ErM fungi in blueberry plants by high-throughput sequencing technology, and it was classified as a mycorrhizal fungus, suggesting that it may play a role in the colonization of mycorrhizal fungi (Yang et al., 2018). *Oidiodendron maius* fungi not only promote plant growth but also induce a large number of root developments. The mechanism is that *Oidiodendron maius* fungi can produce high concentrations of Trp (tryptophan) for IAA synthesis through IPA (indole-3-pyruvate) and IAA to promote mycelium growth. IAA may also induce adventitious root formation in micro-cutting and promote IAA production in plants (Wei et al., 2020). Our results showed that *Om*FC and *Lp*ZL6 colonized the roots of lingonberry and formed mycorrhizal structures (Figure 3E).

After ErM inoculation, the growth status and chlorophyll content of lingonberry changed (Sharma et al., 2020; Baba and Hirose, 2021). Studies have shown that the interaction between mycorrhizal fungi and plants occurs through the secretion of secondary metabolites or the formation of specific symbiotic structures to help the host recruit beneficial colonies and promote water absorption and plant growth and development (Cheng et al., 2021; El-Nashar et al., 2021; Sun et al., 2021). This was the same as our results. After inoculation with *Om*FC and *Lp*ZL6, the stem and root biomass of lingonberry increased significantly. However, plants can also balance endophytic fungi through nutritional distribution, secretion of specific substances and other ways to prevent their excessive proliferation (Liu et al., 2020).

Under drought stress, mycorrhizal fungi can assist plants in coping with water shortage environments, enhance plant water and nutrient absorption, and promote photosynthesis and transpiration (Amiri et al., 2017; Grelet et al., 2017; Ghanbarzadeh et al., 2019). The activation of the plant antioxidative stress system reduces oxidative damage, maintains plant cell stability, promotes plant uptake of nitrogen, phosphorus and other elements, and helps plants resist drought and soil pathogens (Abdel-Salam et al., 2018; Li et al., 2019). This is highly consistent with our results. It is exciting that under drought stress, *Lp*ZL6 inoculation can significantly increase the growth of lingonberry roots, up to 1157.6% (Figure 6B). This result is consistent with in *Rhododendron fortunei*; ErM fungi can significantly increase root biomass (Wei et al., 2020).

**FIGURE 7:**
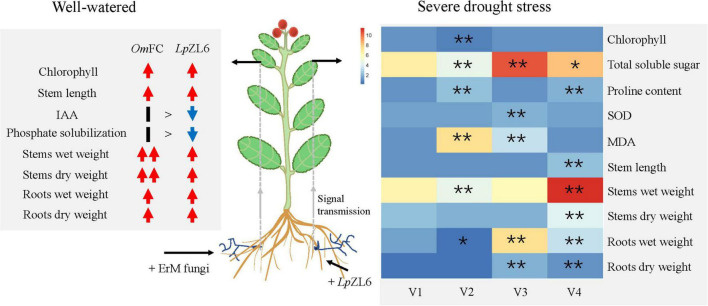
The mechanism of promoting plant growth and alleviating drought stress damage in lingonberry inoculated with ErM fungi. **(Left)** Inoculation with *Om*FC and *Lp*ZL6 increased chlorophyll content, stem and root weight. **(Right)** Inoculation *Lp*ZL6 increased the chlorophyll content, TSS content, and SOD activity of plants through colonization of plant roots and signal transduction, and decreased the proline content and MDA content to alleviate the damage of drought stress on plants. V1: Wild type; V2: + Drought; V3: + Drought + *Lp*ZL6; V4: + *Lp*ZL6.

This study showed that water stress reduced growth parameters (Figure 7). This may be due to water and nutrient absorption and a decrease in plant photosynthetic capacity (Quiroga et al., 2018). Our results showed that inoculation of lingonberry with *Lp*ZL6 improved plant growth parameters under drought stress (Figure 6A). This may be because *Lp*ZL6 fungi provide more water for plants by expanding hyphae and developing roots (Figure 7), resulting in more dry matter production and accumulation in plants (Attarzadeh et al., 2019; Khajeeyan et al., 2019).

Water deficiency stress can reduce the growth of plants and affect the biochemical and physiological processes of plants. In this study, water deficiency led to the accumulation of TSS in plants, which was due to the increased photosynthate and free carbohydrates (Lee et al., 2008). The improvement of TSS in lingonberry may be attributed to increased carbon fixation and enzyme activation (Figure 7). Our study showed that in plants under drought stress, inoculation with *Lp*ZL6 fungi could increase chlorophyll content and photosynthetic activity, thus increasing the content of TSS (Byrne and Mitchell, 2004; Lin et al., 2011; Asrar et al., 2012). Drought-resistant plants can maintain more chlorophyll content under water stress, which is consistent with our experimental results (Fang and Xiong, 2015). Under the condition of water shortage, the chlorophyll content of lingonberry leaves increased, and light energy was used more effectively (Figure 7).

Plants inoculated with *Lp*ZL6 showed reduced proline accumulation in response to severe drought stress (Figure 6H). This was consistent with the results of Ghanbarzadeh et al., where proline content in uninoculated leaves increased significantly under drought stress, while proline accumulation in inoculated fungal plants decreased (Ghanbarzadeh et al., 2020). To protect plants from potential damage under water shortage conditions, proline can become an effective scavenger of ROS to protect the plant cell membrane and other structures from damage (Rapparini and Peñuelas, 2014). In our study, *Lp*ZL6 inoculation reduced the MDA content of lingonberry under drought stress but increased the SOD activity, which had the same results in the study of geranium (Amiri et al., 2015). The above results showed that mycorrhizal fungi could help plants resist water stress by changing the physiological and biochemical processes of plants (Figure 7).

## Data Availability Statement

The datasets presented in this study can be found in online repositories. The names of the repository/repositories and accession number(s) can be found in the article/Supplementary Material.

## Author Contributions

HL, LS, and JZ designed the experiments and wrote the manuscript. HL, CG, and BF analyzed these data. Others participated in the experiments. All authors read and approved the final manuscript.

## Conflict of Interest

The authors declare that the research was conducted in the absence of any commercial or financial relationships that could be construed as a potential conflict of interest. The reviewer AW declared a shared affiliation with the author HS to the handling editor at the time of review.

## Publisher’s Note

All claims expressed in this article are solely those of the authors and do not necessarily represent those of their affiliated organizations, or those of the publisher, the editors and the reviewers. Any product that may be evaluated in this article, or claim that may be made by its manufacturer, is not guaranteed or endorsed by the publisher.
