# Supplementary material for: Lingonberry (Vaccinium vitis-idaea L.) Interact With Lachnum pygmaeum to Mitigate Drought and Promote Growth
Source: Front Plant Sci. 2022 Jun 9;13:920338. doi: 10.3389/fpls.2022.920338 (PMC9218470; doi:10.3389/fpls.2022.920338)
Supplement: Supplementary file 1 [file Table_2.DOCX]

**Table S1.** Primers used in this study.

| Primer name | Primer sequence (5’ to 3’) | Purpose |
| --- | --- | --- |
| ITS1 | TCCGTAGGTGAACCTGCGG | For PCR |
| ITS4 | TCCTCCGCTTATTGATATGC |  |
